# The Inherent Drawbacks of the Pressure to Publish in Health Sciences: Good or Bad Science

**DOI:** 10.12688/f1000research.6809.2

**Published:** 2016-03-24

**Authors:** Ricardo Jorge Dinis-Oliveira, Teresa Magalhães

**Affiliations:** 1 Department of Legal Medicine and Forensic Sciences, Faculty of Medicine, University of Porto, Porto, Portugal; 2 IINFACTS - Institute of Research and Advanced Training in Health Sciences and Technologies, Department of Sciences, Advanced Institute of Health Sciences – North (ISCS-N), CESPU, CRL, Gandra, Portugal; 3 UCIBIO-REQUIMTE, Laboratory of Toxicology, Department of Biological Sciences, Faculty of Pharmacy, University of Porto, Porto, Portugal

**Keywords:** pressure for publication, fraud, peer-review, impact factor, open access and traditional journals

## Abstract

In recent years, there has been a significant increase in the number of scientific publications– it is the era of “hunting the article”. This commentary discusses the drawbacks of the pressure to publish that certainly contribute to the ‘dark side’ of science. In fact, health science career progression greatly relies on the number of scientific publications a researcher has, and in many cases these may be more valorized than the health services provided. Of course, scientific publications help to develop the skills of health care professionals, but as Einstein highlighted “ *not everything that counts can be counted, and not everything that can be counted counts*”.

## Introduction

In recent years, a significant increase in the number of scientific health publications has been registered. Some possible explanations include: (a) the feeling that nowadays “there is no science without being published”, as it corresponds to the permanent record of our research, reputation and “immortality”; (b) the author’s “motivation” to publish due to their need to obtain funding and further their career; (c) the perception that to publish is the individual or team effort to ensure the wide sharing of results; on the other hand, to not publish may suggest that the author is not committed to sharing knowledge and, in some cases, wishes to avoid scientific discussion with peers.

Perhaps in the past it was more difficult to publish than it is at the present. Database access to publications was scarce or nonexistent, and the cycle of publication was time consuming without an online platform. Nevertheless, the competition to publish was not as aggressive, the impact factor and the h index were not a concern, and scientists were not constantly scrutinized according to their publication records/numbers. Nowadays, these aspects have become new worries, increased by new ethical and conflict of interest issues. Thus, to publish is both almost compulsory (a “question of survival”) and simultaneously a very hard task; we are in the era of “hunting the article”, which in some cases may promote fraud and corruption.

How could we explain the 1.7 to 1.8 million articles in 2013 in journals with peer-review (a supposed certificate of quality), or approximately one article every 18 seconds? ^1^ Moreover, and in spite of significant innovation, some studies suggest that the average peer-review takes approximately 10.9 and 6.5 hours for young *versus* experienced reviewers, respectively ^2^. In addition, according to Mabe ^3^ the number of new peer reviewed journals and articles published annually has been growing at a very steady rate of about 3–3.5% per year for over three centuries, but in the last few years this increase has become more pronounced.

Publishing is also a business and some authors even suggest that it is becoming pathological, with psychological and legal implications ^4^. Indeed, the area of scientific publication moves many materials and human resources. The *International Association of Scientific, Technical and Medical* (STM) *Publishers* that holds 66% of the publication market and each year publishes nearly two-thirds of all journal articles, handled $9.4 billion in 2011 (up from $8 billion comparatively in 2008) solely in scientific journals, and employs about 110,000 people. Although the United States of America (USA) continues to dominate the global production of research papers, significant growth has also been registered for China and East Asia.

This commentary aims to highlight some relevant aspects of scientific publications that all health care providers and researchers, as well as medical students, should better understand in order to avoid publication misconduct.

## Concerns in selecting scientific journals

In traditional journals, articles are usually behind a ‘pay-wall’, meaning that readers must have a subscription or pay a fee to read content. In opposition to this traditional method, the number of open access (OA) journals have been increasing in the last few years ^5^. OA journal models comprise two main approaches:

### 1. OA publishing or the “Gold route”

This model of OA publishing has been increasing in popularity ^6^ and represents direct publication in OA journals, with the majority requiring payment of the cost of publication. Nevertheless, some are sponsored, which means that the author does not pay or some institutions may have an agreement with publishers ( *i.e.* the OA membership). A hybrid or optional variant exists ( *i.e.* only part of the article is immediately OA and, if wanted, the author may pay for the full article to be OA);

### 2. Delayed free access and self-archiving, or the “Green route”

In this case, some traditional journals, after an embargo (necessary to recover the investment by publishers), allow the publication of some articles in free repositories ( *e.g.* MEDLINE, PsyDok). For free access after the embargo these journals may require the payment of article processing charges.

There are around 10,128 fully OA journals listed on the *Directory of Open Access Journals* ^7^. This publication model is important for future citation and therefore to maximize the impact of research. The clients of the publishers are the authors and not the readers, but the exaggeration in the number of journals and articles may even disquiet the best proponents of the OA model. For example, in 2013 *PLOS ONE* published approximately 31,500 articles (a staggering increase from 138 articles in 2006), meaning almost one in every 60 PubMed articles if from *PLOS ONE* ^8^. This “megajournal” structure is widely accepted as one of the most trusted OA journals, practicing a very broad scope and a rapid “non-selective” peer review based on “soundness not significance” ( *i.e.* selecting papers on the basis that science is soundly conducted rather than more subjective criteria of impact, significance or relevance to a particular community) ^2^. Currently, Brazil is the country with the most OA journals after the USA; more than one thousand. That abundance was recently described as “a plague of Brazilian science: articles of second class” ^9^.

In our opinion one of the best papers alerting the community to this problem was published by Bohannon ^10^. The author investigates the peer-review process of the fee-charging OA journals. Between January and August 2013, Bohannon submitted obvious false articles to 304 scientific journals. In the author’s opinion the article should have been immediately rejected by the editors and reviewers, but 60% of journals accepted it. The article was based on multiple combinations of the “X molecule of species Y lichens that inhibits the growth of cancer cells Z”. For each article, Bohannon even faked the authors and affiliations. All articles essentially concluded that those molecules had therapeutic activity in several cancer types. The study also showed a map of the geographical location of the editors and publishers and their bank accounts. The author concluded that any reviewer with basic knowledge of chemistry and with skills to analyze an elementary graph should have immediately detected the article’s faults. He concluded that “it was easy to jump directly from the test tube to the clinic”.

Subsequently, Hvistendahl ^11^ reported that in China, a black market has been developed by some agencies to allow authors to write articles without a need to perform experimental work. The prices depend on whether the person paying wishes to be listed as the primary writer, or as merely a co-author, or even as just one of the team members. Authors suggested that this black market may have contributed to China’s rapid growth in the number of Science Citation Index (SCI) articles, with China’s contribution now the second largest, after the USA. Curiously, a running joke in China offered another meaning for SCI: *“Stupid Chinese Idea”* ^12^.

Furthermore, researchers are bothered with several invitations to review or submit articles that are not within their main area of research, and the same invitations are distributed to many email accounts. Perhaps also sharing the same annoyance at this state of affairs, Jeffrey Beall in August 2012 published the first edition of the “Criteria for Determining Predatory Open-Access Publishers”. These criteria were the start point to generate a list of potential, possible, or probable predatory OA scholarly publishers.

Similar problems may also occur with traditional journals, even peer-reviewed. The question is no longer if we should have OA journals. In fact, they are a very positive way to share scientific knowledge for all researchers, and many possess great credibility. The question now is what journals we should choose and how to assess their credibility. To help to solve this problem, it is now possible for readers to perform a continuous re-reviewing of published articles as it occurs for *F1000Research*. Through the readers’ opinions, journal credibility will be scrutinized.

## Constraints of the peer review cycle

Peer review is a methodological evaluation on the soundness of the topic, originality, methodology, results and conclusions highlighted by the author and the authorities cited. Although it cannot generally assure that the data is truthful or not, peer review unquestionably increases the quality of most manuscripts. Nevertheless, this procedure is sometimes slow, expensive, profligate, subjective, prone to bias, poor at detecting gross defects, and almost useless in detecting fraud ^13^.

Typically pre- ( *e.g.* double and single-blind, and open) or post-publication peer review is practiced. *Double-blind peer review* is more common in the humanities and social sciences than in the exact sciences. Although the identity of the reviewers is not disclosed and *vice-versa* (which removes the potential influence of the involved countries, institutions or eventual personal conflicts), the process sometimes fails, because the method, style of writing, acknowledgments and abuse of self-citations can suggest the source. In *single-blind peer review* the author is unaware of the identity of the reviewers; it is the most common process, especially in life sciences and is useful for the reviewer in order to consult previous authors’ works. By the same reasons listed for the double-blind peer-review, the process may sometimes fail. Although less common than the previous peer reviews, the *open peer review* process is increasing: nevertheless, it results in less acceptances to review since reviewers are afraid of being identified if commenting negatively on the article ^14^. Nevertheless, it is expected that reviewers produce better revisions and avoid offensive or rude words. The name of the reviewers and comments may be published alongside the article as it occurs in the *British Medical Journal*.

Once the pre-publication peer review process is completed, the decision to publish is made by the journal editor on the advice of the reviewers. The editor of a journal is usually an independent, leading expert in his/her field appointed and sometimes financially supported by the publisher. An apparent misuse of editorial privileges was practiced by the editor of “Chaos, Solitons and Fractals” (a theoretical-physics journal), who was criticized by using its pages to publish numerous articles written by himself ( *e.g.* 36 papers in the December 2008 issue) ^15^.

Finally, in *post-publication peer review*, the quality is assessed by the sapient of crowds and readers can also judge the quality of the review process. This peer review is outside the editor’s monopoly and journals usually also provide a discussion forum about the article in order for researchers to comment on and read other’s comments. Similar to the *British Medical Journal*, referee reports and names are published alongside the article, together with the authors' responses to raised points. It is believed that both invited and un-invited ( *i.e.*, commenting) post publication peer review helps to increase the quality of the final publication and it is certainly more transparent. *F1000Research* follows this model and advocates that time is not lost in reviews, since each article is only subjected to editorial verification and within approximately one week is published online already formatted and can be read and cited ( http://f1000research.com/about).

## Limitations of the impact factor

In 1960, the *Institute for Scientific Information* (ISI) was founded by Eugene Garfield and later he proposed the impact factor (IF) as a tool for journal evaluation by librarians to help them with journal purchasing decisions ^16^. The ISI was purchased by *Thomson Scientific & Healthcare* in 1992 becoming the “Thomson ISI”. IF it is not a perfect tool to measure the prestige of the journal, but there is no better. Falagas and colleagues performed a very interesting discussion on metrics for scientific journals as well as for researchers ^17^. Some biases of the IF include: (a) it is not statistically representative of individual articles; (b) review articles (often highly cited) greatly skew the results; (c) extensive articles always have many citations and yield a high IF; (d) it includes self-citations; (e) books do not count for citations; (f) databases primarily index articles in English (but the IF also exists for non-English journals) and are dominated by USA publications; (g) it is dynamic since it depends on fluctuations of research in a given area; (h) paid access of some journals; (i) journals with tight scope tend to have low IF; (j) it does not take into account the subjects variability ( *e.g.*, immunology and cancer are usually highly cited); (k) editors aware of the importance of the IF tend to accept articles that may be highly cited and to reduce the number of articles accepted; (l) the absence of an IF in a given journal can result in low submissions; (m) it is only applied to ISI journals; (n) one highly cited article can boost the IF in a given journal ( *e.g.*, the impressive IF of 49.9 for *Acta Crystallographica Section A: Foundations of Crystallography*; the primary cause of this high impact factor was a single feature article by Sheldrick ^18^).

## Fraud in life sciences

Nowadays, there is rising interest in research and publication ethics. Proof of that is the increased importance of organizations such as the *Committee on Publication Ethics* (COPE) and the development of software to detect plagiarism. Although, the number of journal article retractions has grown in the last decade ^19^, it is the general consensus that this may be the result of increased awareness rather than misconduct ^2^. Nevertheless, several fraudulent/misconduct cases have been made publicly available.

Andrew Jeremy Wakefield, a former British surgeon and researcher, published a fraudulent study in 1998 claiming that there was an association between the administration of the measles, mumps and rubella vaccine, and the development of autism and Crohn’s disease ^20, 21^.

The German physicist Jan Hendrik Schön, starred a scandal related to semiconductors that triggered a series of retractions, six of them out of *Science* ^22^. Hwang Woo-suk ( *i.e.* the pride of South Korea) was sentenced to two years in prison with suspended sentence after distorting the results published in two *Science* articles related to cloning of human embryonic stem cells ^23, 24^.

More recently in January 2014, Haruko Obokata, a young researcher in Japan published in *Nature*, showed stem cells can now be made quickly just by dipping blood cells into acid ^25, 26^. On June 2014, Obokata agreed to retract both the papers and 2 months later Obokata’s mentor and co-author, Yoshiki Sasai, committed suicide by hanging. Although investigation cleared him of misconduct, he was not free of critiques for inadequate supervision of Haruko Obokata.

In 2005, the researchers David Mazières and Eddie Kohler designed an anecdote manuscript, to send in response to unsolicited congress invitations. Later in 2014, Peter Vamplew, an associate professor at the Federation University Australia School of Engineering and Information Technology, after receiving a spam email from the *International Journal of Advanced Computer Technology* (classified as predatory OA on Beale’s list’), forwarded Mazières’ and Kohler’s old paper as a response. The journal’s peer-review process classified the manuscript as “excellent” and accepted it for publication ^27^. At the end, the manuscript was not actually published since Vamplew declined to pay the article processing charge. Acceptance of a paper consisting entirely of 863 repetitions of “Get me off your fucking mailing list” has led commenters to question whether the enterprise is more interested in collecting publication fees than in contributing first-rate peer reviewed articles to the advancement of computer science ^27^.

 Some epic/record examples of fraud were practiced by Joachim Boldt and Yoshitaka Fujii. Joachim Boldt is a German anesthesiologist who was dismissed of his professorship and is under criminal investigation for having allegedly faked of up to 90 research studies ^28^. Yoshitaka Fujii is a Japanese researcher in anesthesiology, who in 2012 was found to have fabricated data in at least 172 scientific papers over the past 19 years, setting what is believed to be a record for the number of papers by a single author requiring retractions ^29, 30^. Many of the listed co-authors did not know they were authors and their signatures had been forged in the copyright transfer.

## Concluding remarks

Being a scientist is a stimulating and gratifying task, but full of difficulties. Scientists do not have schedules and need to fight for financial support; their careers grow exponentially when they publish (especially in high IF journals) and their job security frequently depends on the number of publications. But first of all, scientists are human beings, with families, needs and emotions. Therefore, the motivation to be successful and remain employed may increase the risk of involvement in scientific fraud or even corruption. This may lead authors, journals and publishers to lose their credibility. Particularly problematic is the impact of fraud in areas that have significant impact on the health, safety and welfare of the world’s population, as are the cases of life and health sciences. Moreover, scientific research slows since it is necessary to spend more time confirming published results. Taradi and colleagues show that over 90% of the medical students of Croatia admitted to engaging in education dishonesty and over 78% engaging in academic misconduct ^31^. Indeed, fraud can help the scientist rise, but also fall rapidly.

We need to change the paradigm of scientific research. We cannot grow at all costs. It is also relevant to make peer review more transparent to produce publications that are more genuine and free from bias. The increase in publications, research studies split into multiple publications (rather than single, longer articles), the proliferation of journals, the ways in which academic promotion fosters this proliferation of publications, and the ways in which these changes can encourage bad or even fraudulent science are important topics that will certainly dictate the future of life and health sciences research.

Fortunately, fraud and corruption are punctual cases. Nevertheless, we will face problematic times if financial issues superimpose the ethics in publishing. This “crisis” may be an opportunity and challenge to reflect on these topics.

## References

[1] Björk BC Roos A Lauri M: Scientific journal publishing: Yearly volume and open access availability. *Information Research.* 2009;14(1). Reference Source

[2] Ware M Mabe M: The stm report: an overview of scientific and scholarly journal publishing.3rd ed. Netherlands: International Association of Scientific, Technical and Medical Publishers.2012 Reference Source

[3] Mabe M: The growth and number of journals. *Serials: The Journal for the Serials Community.* 2003;16(2):191–7. Reference Source

[4] Buela-Casal G: Pathological publishing: A new psychological disorder with legal consequences? *Eur J Psychol Appl L.* 2014;6(2):91–7. 10.1016/j.ejpal.2014.06.005

[5] Van Noorden R: Open access: The true cost of science publishing. *Nature.* 2013;495(7442):426–9. 10.1038/495426a 23538808

[6] Van Noorden R: Britain aims for broad open access. *Nature.* 2012;486(7403):302–3. 10.1038/486302a 22722166

[7] Number of listed open access journals.2015 Reference Source

[8] Graham K: Thanking our peer reviewers. *Plos One Community Blog.* 2014 Reference Source

[9] Uma praga da ciência brasileira: os artigos de segunda. *Veja.* 2014 Reference Source

[10] Bohannon J: Who's Afraid of Peer Review? *Science.* 2013;342(6154):60–5. 10.1126/science.342.6154.60 24092725

[11] Hvistendahl M: China's publication bazaar. *Science.* 2013;342(6162):1035–9. 10.1126/science.342.6162.1035 24288313

[12] Sun Q Xin Q Wei L: “Science Citation Index Worship” in China. *Iran J Public Health.* 2013;42(8):921–2. 26056648 PMC4441925

[13] Smith R: Opening up BMJ peer review. *BMJ.* 1999;318(7175):4–5. 10.1136/bmj.318.7175.4 9872861 PMC1114535

[14] Ware M Monkman M: Peer Review in scholarly journals: an international study into the perspective of the scholarly community. Bristol: Mark Ware Consulting.2008 Reference Source

[15] Schiermeier Q: Self-publishing editor set to retire. *Nature.* 2008;456(7221):432. 10.1038/456432a 19037282

[16] Garfield E: Citation analysis as a tool in journal evaluation. *Science.* 1972;178(4060):471–9. 10.1126/science.178.4060.471 5079701

[17] Falagas ME Kouranos VD Arencibia-Jorge R: Comparison of SCImago journal rank indicator with journal impact factor. *FASEB J.* 2008;22(8):2623–8. 10.1096/fj.08-107938 18408168

[18] Sheldrick GM: A short history of SHELX. *Acta Crystallogr A.* 2008;64(Pt 1):112–22. 10.1107/S0108767307043930 18156677

[19] Van Noorden R: Science publishing: The trouble with retractions. *Nature.* 2011;478(7367):26–8. 10.1038/478026a 21979026

[20] Wakefield AJ Murch SH Anthony A: Ileal-lymphoid-nodular hyperplasia, non-specific colitis, and pervasive developmental disorder in children. *Lancet.* 1998;351(9103):637–41. 10.1016/S0140-6736(97)11096-0 9500320

[21] Godlee F Smith J Marcovitch H: Wakefield’s article linking MMR vaccine and autism was fraudulent. *BMJ.* 2011;342:c7452. 10.1136/bmj.c7452 21209060

[22] Bao Z Batlogg B Berg S: Retraction. *Science.* 2002;298(5595):961. 10.1126/science.298.5595.961b 12416506

[23] Hwang WS Ryu YJ Park JH: Evidence of a pluripotent human embryonic stem cell line derived from a cloned blastocyst. *Science.* 2004;303(5664):1669–74. 10.1126/science.1094515 14963337

[24] Hwang WS Roh SI Lee BC: Patient-specific embryonic stem cells derived from human SCNT blastocysts. *Science.* 2005;308(5729):1777–83. 10.1126/science.1112286 15905366

[25] Obokata H Wakayama T Sasai Y: Stimulus-triggered fate conversion of somatic cells into pluripotency. *Nature.* 2014;505(7485):641–7. 10.1038/nature12968 24476887

[26] Obokata H Sasai Y Niwa H: Bidirectional developmental potential in reprogrammed cells with acquired pluripotency. *Nature.* 2014;505(7485):676–80. 10.1038/nature12969 24476891

[27] Bogus journal accepts profanity-laced anti-spam paper. *Scholarly Open Access.* 2014 Reference Source

[28] Millions of surgery patients at risk in drug research fraud scandal. *The Telegraph.* 2011 Reference Source

[29] A New Record for Retractions? (Part 2). *Scienceinsider.* 2012 Reference Source

[30] Anesthesiologist Fabricates 172 Papers.2012 Reference Source

[31] Kukolja Taradi S Taradi M Knežević T: Students come to medical schools prepared to cheat: a multi-campus investigation. *J Med Ethics.* 2010;36(11):666–70. 10.1136/jme.2010.035410 20797977

