# Supplementary material for: The Inherent Drawbacks of the Pressure to Publish in Health Sciences: Good or Bad Science
Source: F1000Res. 2016 Mar 24;4:419. Originally published 2015 Jul 29. [Version 2] doi: 10.12688/f1000research.6809.2 (PMC4648225; doi:10.12688/f1000research.6809.2)
Supplement: Peer review history

# Peer review: Referee response for version 1

**DOI:** 10.5256/f1000research.7321.r10709

**Published:** 2015-10-15

**Authors:** Rita Ferreira, Rui Vitorino

**Affiliations:** 1 Department of Chemistry, University of Aveiro, Aveiro, Portugal

We found the opinion article of Dinis-Oliveira and Magalhães very pertinent as it highlights the pressure that researchers face to show scientific production indicators. Such pressure for “hunting the article”, one of the most used indicators of scientific production, clearly distorts the ideal of any young scientist, to make scientific discoveries with relevance to humankind. It would be interesting to add some discussion on the association between funding and scientific production, as it is not always straightforward.

We have read this submission. We believe that we have an appropriate level of expertise to confirm that it is of an acceptable scientific standard.

# Peer review: Referee response for version 1

**DOI:** 10.5256/f1000research.7321.r10656

**Published:** 2015-10-02

**Authors:** Frederico Pereira

**Affiliations:** 1 Laboratory of Pharmacology and Experimental Therapeutics, University of Coimbra, Coimbra, Portugal

This opinion article timely captures the air du temps` concerning the overwhelming pressure to publish in health sciences. The authors specifically delved into the core of concerns about selecting scientific journals, constrains of peer review processes and limitations of impact factor. Finally I should stress the title provides an appropriate summary of the content of the paper and warrants readership.

I have read this submission. I believe that I have an appropriate level of expertise to confirm that it is of an acceptable scientific standard.
